# Factors associated with the nutritional status of the older population in a selected area of Dhaka, Bangladesh

**DOI:** 10.1186/s12877-021-02068-2

**Published:** 2021-03-05

**Authors:** K. M. Thouhidur Rahman, Md. Khalequzzaman, Fahmida Afroz Khan, Shahrin Emdad Rayna, Sharraf Samin, Md. Hasan, Syed Shariful Islam

**Affiliations:** Department of Public Health and Informatics, Bangabandhu Sheikh Mujib Medical University, Room # 309, Block # B, Shahbag, Dhaka, 1000 Bangladesh

**Keywords:** Malnutrition, Older population, Mini nutritional assessment, Geriatric depression scale-short form, Geriatric Oral health assessment index, Bangladesh

## Abstract

**Background:**

Globally, older population (aged ≥60 years) comprise 11% of the total population, and 23% of them are malnourished. Lack of knowledge and education, adverse dietary habits, depression or psychological disorders, poor oral and dental health, disability, and diseases are the reported factors responsible for malnutrition among them. Geriatric people comprise 7.5% of the total population of Bangladesh, and almost a quarter are malnourished. But there is scarce data on the factors associated with the nutritional status of the older population in Bangladesh.

**Methods:**

A cross-sectional study was conducted among 125 older individuals (male 59, female 66) living in three villages of Uttarkhan, Dhaka, Bangladesh, to identify the factors associated with their nutritional status. The Mini Nutritional Assessment scale, Geriatric Depression Scale-Short Form, and Geriatric Oral Health Assessment Index were used for assessing the nutritional status, mental health status, and oral health quality of the respondents, respectively. Information on socio-demographic characteristics, comorbidities and dietary factors, and food behaviors were gathered by a pretested semi-structured questionnaire. Ethical approval was obtained from the Institutional Review Board of Bangabandhu Sheikh Mujib Medical University, Dhaka, Bangladesh.

**Results:**

The participants’ mean age was 67.9 ± 7.1 years. Most of them (53.6%) had no formal education. Among the respondents, 22.0% male and 28.8% female were malnourished. The proportion of malnourished and at risk of malnutrition among the respondents living without a partner were 28.6 and 65.3%, respectively. A significantly (*p* < 0.05) higher odds of having depression (OR 15.6; 95% CI 3.1–78.1), poor oral health (OR 7.3; 95% CI 1.3–41.8), and no formal education (OR 6.5; 95% CI 1.3–32.1) was observed among the malnourished respondents. Though it was not statistically significant, among the malnourished, 31.3, 25.0 and 25.0% avoided highly oily food, beef/mutton, and sugary food, respectively.

**Conclusions:**

More than two-thirds of the older population were malnourished or at risk of malnutrition, where the female respondents were more vulnerable. Depression, inadequate oral health, and lack of education were negatively associated with the nutritional status of the older population.

## Background

In recent times, nutrition-related health problems, disease morbidity, and the physical inability of the older population are coming to the surface at a higher rate [1]. Among the affected, most of the population are from developing countries [2]. About one-third of the people living in these countries are malnourished, consisting of a significant proportion who are older (aged 60 years or more) [3, 4].

The social burden of malnutrition seems to be much more characterized by a massive shift in deterioration of the physical and psychological components of health [5]. Most of the malnourished older people living in the community or nursing homes are at risk of developing different nutrition-related complications, leading to the inability to live on their own and be dependent on others [5, 6]. Various comorbidities and nutritional status of older individuals are intermingled as they are more prone to chronic diseases, which leads to further malnutrition, making it a vicious cycle [7].

Globally, many factors are associated with malnutrition of the older population [8]. Gender, marital status, education, and expenditure of the family [7–10], mental health status [11], oral health quality [12], comorbidities [13–15], and food behavior [6, 16] are some crucial factors that are causing malnutrition in that age group. Identifying the factors responsible for the nutritional status of the older population and preventing them by timely social interventions may result in a better health prognosis and reduce the malnutrition burden [17, 18].

In Bangladesh, 7.5% of the total population are of older age group [19, 20]. Among them, 26.0% are malnourished, and 62.0% are at risk of malnutrition [7, 21].

However, there are no comprehensive data regarding the factors associated with the nutritional status of the older population in Bangladesh. Thus, this study aimed to identify the factors which are responsible for malnutrition of the older population.

## Methodology

### Study design and population

This cross-sectional study was conducted by the Department of Public Health and Informatics (DPHI), Bangabandhu Sheikh Mujib Medical University (BSMMU) in Uttarkhan Upazila, Dhaka, Bangladesh, from November 2019 to February 2020. As referred by the United Nations, people aged 60 years or more were considered as older population [4], and respondents of that age group living in three villages of Uttarkhan, Dhaka were the study population.

### Sampling method

A sampling frame was formed from a previous study conducted by DPHI, BSMMU in three villages of Uttarkhan, Dhaka (data not yet published) consisting of 251 older residents. From that sampling frame, due to time and financial constraint, a total of 180 samples were purposively selected and contacted for interview. Among them, 125 agreed to participate in this study, 26 participants refused, and 29 participants were not found in the designated address. The response rate of participants for this study was 69.4%.

### Ethics

This study was conducted according to the Declaration of Helsinki and was performed after getting ethical clearance from the Institutional Review Board of Bangabandhu Sheikh Mujib Medical University (BSMMU). During data collection, eligible subjects were contacted by the interviewers ensuring confidentiality. They received an informed consent form with the details of the research, rights regarding their participation, and withdrawal at any time. They were informed that anonymity will be maintained. From all the subjects, informed written consent was taken. Approval for the use of certain instruments for physical measurements in the study was also taken before data collection.

### Data collection tool

A pretested semi-structured questionnaire used in the face to face interviews for data collection was adapted from the Mini Nutritional Assessment (MNA) Scale [22], the Geriatric Depression Scale Short Form (GDS-SF) [23], Charlson Comorbidity Index [24, 25], and the Geriatric Oral Health Assessment Index (GOHAI) [26]. To make it reliable and valid, an expert first translated the tools into Bengali. Then the Bengali versions were back translated into English by a different person and compared for consistency. The consistent tools were then sent for pretesting in Rayerkhola, Dhaka, among 15 participants. After pretesting, necessary modifications were done to make it culturally consistent. Anthropometric measurements were taken by measuring tapes and regularly calibrated bathroom weighing scales**.**

#### Outcome measurement: nutritional status

The nutritional status assessment was done by using the MNA scale, where six screening questions and twelve assessments (i.e., height, weight, mid-arm circumference, calf circumference) were taken into consideration. The scores assigned for individual responses were according to the MNA questionnaire [22]. The total score was 30, and 0 to < 17 was categorized as malnourished, 17 to 23.5 as at risk of malnutrition, and 24 to 30 as well-nourished [22]. In previously conducted study, the sensitivity and specificity of the MNA scale was found as 96 and 98%, respectively, with a positive predictive value (PPV) of 97% [27]. Soysal et al. (2019) reported the internal consistency (Cronbach alpha coefficient) of MNA scale as 0.70 [28]. For this study, the internal consistency and reliability for MNA was checked where the Cronbach alpha coefficient was 0.71.

#### Major factors measurements

##### Socio-demographic characteristics

Socio-demographic data regarding age, gender, religion, living without partner (spouse), education, occupation, number of family members, and socio-economic status were collected from the participants. Besides these, information on whether the participant lived alone or with a partner was also collected.

##### Mental and oral health status

GDS-SF and GOHAI were used for assessing the mental health status and oral health quality of the study population, respectively. The total score for GDS-SF ranged from 0 to 15 [23], and for GOHAI, it was 0 to 60 [26]. Mental health assessment and oral health assessment were done by adding the scores of responses against individual questions. Then the scores were categorized and presented in tabular form. The total score of 0 to 4 in GDS-SF was considered as normal mental health, and 5 to 15 was considered as depressed [23]. In a validation study, the sensitivity, specificity and PPV, of GDS-SF in determining depression were 92, 91 and 76%, respectively with an internal consistency (Cronbach alpha) of 0.92 [29]. In this current study, the Cronbach alpha of GDS-SF was 0.87. According to GOHAI, scores < 51 were treated as poor oral health, 51 to 56 as moderate oral health, and 57 to 60 were considered good oral health quality [26]. GOHAI is an acceptable tool with good internal consistency and reliability with a Cronbach alpha of 0.79 [30]. This current study found the Cronbach alpha for GOHAI as 0.87.

##### Comorbidities and dietary factors

Regarding comorbidities of the participants, the prevalence of diseases like arthritis/rheumatic diseases, chronic kidney diseases, chronic obstructive pulmonary disease (COPD)/asthma/chronic cough, diabetes, eye diseases, hypertension, myocardial infarction (MI)/heart failure, neurological disorders (tingling/weakness of limb), peptic ulcer disease and stroke/paralysis were recorded by checking their medical documents.

##### Food behaviors

Food behaviors regarding egg, milk/milk products, sugary foods, beef/mutton, allergic foods, and highly oily foods consumption by the respondents were recorded by asking questions.

### Statistical analysis

Socio-demographic characteristics are presented by descriptive statistics. Data related to prevailing comorbidities and food behavior are presented in frequency and percentage. Chi-square test, and Fisher-Freeman-Halton test was performed for categorical variables, and for continuous variables, Analysis of Variance (ANOVA) was used to analyze their association. Multinomial logistic regression was conducted to find out the association between nutritional status and major factors. Results are presented by adjusted odds ratio (OR) with 95% confidence interval (CI). Test results are considered statistically significant if the *p*-value is < 0.05. Data cleaning and detailed analysis were performed using the IBM SPSS software version 25.0.

## Results

**Fig. 1:**
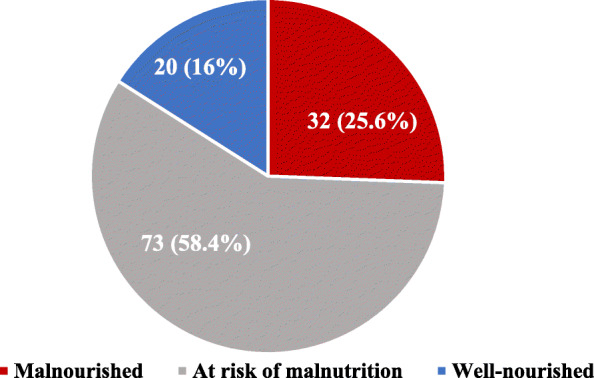
Nutritional status of the respondents

A total of 125 respondents completed the interview, and among them, 25.6% were malnourished, and 58.4% were at risk of malnutrition (Fig. 1).

Table 1 shows the socio-demographic characteristics of the respondents. The mean age for well-nourished, at risk of malnutrition, and malnourished were 64.7 ± 3.9, 68.2 ± 7.7, and 69.6 ± 6.8 years, respectively, and the difference was statistically significant (*p* = 0.021). The proportion of malnutrition and at risk of malnutrition were higher in females (28.8 and 62.1%) than those of males (22.0 and 54.3%). Among the subjects, 28.6 and 65.3% were malnourished and at risk of malnutrition, respectively, who were living without partners. A significantly (*p* = 0.025) higher proportion of malnutrition was observed among respondents with no formal education (32.8%) compared with education up to primary (20.0%) and secondary level (15.2%).

**Table 1:** Socio-demographic characteristics of the respondents categorized by nutritional status

Variables	Malnourished (***n*** = 32)	At risk of malnutrition (***n*** = 73)	Well-nourished (***n*** = 20)	***P***-value
Age (in years)				0.021
Mean ± SD	69.6 ± 6.8	68.2 ± 7.7	64.7 ± 3.9	
Gender n (%)				0.08
Male	13 (22.0)	32 (54.3)	14 (23.7)	
Female	19 (28.8)	41 (62.1)	6 (9.1)	
Living without partner n (%)				0.053^a^
Yes	14 (28.6)	32 (65.3)	3 (6.1)	
No	18 (23.7)	41 (53.9)	17 (22.4)	
Education status n (%)				0.025^a^
No formal education	22 (32.8)	39 (58.2)	6 (9.0)	
Up to primary education	5 (20.0)	17 (68.0)	3 (12.0)	
Secondary and above	5 (15.2)	17 (51.5)	11 (33.3)	
Occupational status n (%)				0.298^a^
Employed	3 (16.7)	9 (50.0)	6 (33.3)	
Homemaker	11 (23.4)	30 (63.8)	6 (12.8)	
Unemployed/Retired	18 (30.0)	34 (56.7)	8 (13.3)	

^a^Fisher–Freeman–Halton’s Test

The mental and oral health status of the respondents by nutritional status is presented in Table 2**.** Significantly (*p* < 0.001), a higher proportion of malnutrition was observed among the respondents who had depression (39.3%) than those with normal mental health (12.5%). About one-third (29.6%) of the respondents with poor oral health quality were malnourished than those of good oral health quality (12.5%), and the difference was statistically significant (*p* = 0.002).

**Table 2:** Mental and oral health status of the respondents categorized by nutritional status

Variables	Malnourished (***n*** = 32)	At risk of malnutrition (***n*** = 73)	Well-nourished (***n*** = 20)	***P***-value
Depression n (%)				< 0.001^a^
Depressed	24 (39.3)	34 (55.7)	3 (4.9)	
Normal mental health	8 (12.5)	39 (60.9)	17 (26.6)	
Oral health status n (%)				0.002^a^
Poor	24 (29.6)	51 (63.0)	6 (7.4)	
Moderate	5 (25.0)	11 (55.0)	4 (20.0)	
Good	3 (12.5)	11 (45.8)	10 (41.7)	

^a^Fisher–Freeman–Halton’s Test

Table 3 shows the food behavior and comorbidities of the respondents by nutritional status. A quarter or more of the malnourished respondents avoided highly oily food (31.3%), beef/mutton (25.0%), and sugary foods (25.0%). Among the malnourished respondents, 40.6% avoided any of the observed food items (data not shown). The majority of the malnourished respondents had eye diseases (68.8%), peptic ulcer disease (62.5%), and neurological diseases (50.0%).

**Table 3:** Food behavior and comorbidities of the respondents categorized by nutritional status

Variables	Malnourished (***n*** = 32)^b^	At risk of malnutrition (***n*** = 73)^b^	Well-nourished (***n*** = 20)^b^	***P***-value
Avoid any kind of food items	13 (40.6)	25 (34.2)	9 (45.0)	0.641
Items avoided				
Highly oily foods	10 (31.3)	11 (15.1)	5 (25.0)	0.137^a^
Beef/Mutton	8 (25.0)	8 (11.0)	3 (15.0)	0.189^a^
Sugary foods	8 (25.0)	8 (11.0)	4 (20.0)	0.15^a^
Allergic foods	5 (15.6)	7 (9.6)	3 (15.0)	0.592^a^
Milk/Milk products	3 (9.4)	7 (9.6)	3 (15.0)	0.719^a^
Egg	2 (6.3)	5 (6.8)	2 (10.0)	0.792^a^
Comorbidities				
Eye diseases	22 (68.8)	43 (58.9)	11 (55.0)	0.538
Peptic ulcer disease	20 (62.5)	45 (61.6)	10 (50.0)	0.607
Neurological diseases	16 (50.0)	25 (34.2)	4 (20.0)	0.08^a^
Hypertension	15 (46.9)	31 (42.5)	9 (45.0)	0.912
Arthritis/Rheumatic diseases	14 (43.8)	39 (53.4)	7 (35.0)	0.294
Stroke/Paralysis	8 (25.0)	8 (11.0)	1 (5.0)	0.1^a^
MI/Heart failure	7 (21.9)	15 (20.5)	5 (25.0)	0.911
Diabetes	6 (18.8)	15 (20.5)	4 (20.0)	1.0^a^
COPD/Asthma/Chronic cough	6 (18.8)	10 (13.7)	1 (5.0)	0.408^a^
Chronic kidney diseases	4 (12.5)	8 (11.0)	1 (5.0)	0.845^a^

^a^Fisher–Freeman–Halton’s Test

^b^All data is shown as number (%)

Association of nutritional status with selected factors by multinomial logistic regression are depicted in Table 4. For the regression process, the well-nourished category was taken as a reference for comparing with at risk of malnutrition and malnourished group. A significantly higher odds of having malnutrition was observed among the respondents with depression (OR 15.6; 95% CI 3.1–78.1, *p* = 0.001), poor oral health quality (OR 7.3; 95% CI 1.3–41.8, *p* = 0.026) and no formal education (OR 6.5; 95% CI 1.3–32.1, *p* = 0.022) than the respondents with normal mental health, good oral health quality and education up to secondary and above, respectively. The study also found that the odds of being in the ‘at risk of malnutrition’ group is higher among the respondents with depression (OR 4.5; 95% CI 1.1–19.0, *p* = 0.04) and poor oral health quality (OR 5.4; 95% CI 1.5–20.2, *p* = 0.011), which was statistically significant.

**Table 4:** Association of nutritional status with selected factors^c^

Variables^a,b^	***Malnourished***		***At risk of malnutrition***	
	Adjusted OR (95% CI)	***P***-value	Adjusted OR (95% CI)	***P***-value
Education status				
Secondary and above	Ref		Ref	
Up to primary education	3.4 (0.5, 25.5)	0.233	3.3 (0.7, 16.3)	0.150
No formal education	6.5 (1.3, 32.1)	0.022	3.3 (0.8, 13.3)	0.058
Mental health status				
Normal mental health	Ref		Ref	
Depressed or altered mental health	15.6 (3.1, 78.1)	0.001	4.5 (1.1, 19.0)	0.04
Oral health status				
Good	Ref		Ref	
Moderate	4.6 (0.5, 37.7)	0.155	2.6 (0.5, 12.7)	0.241
Poor	7.3 (1.3, 41.8)	0.026	5.4 (1.5, 20.2)	0.011

^a^Dependent Variable: Nutritional status (well-nourished was taken as the reference category)

^b^Independent variables: Age, gender, marital status, education status, occupation status, mental health status, and oral health status

^c^Results of age, gender, marital status, and occupation status were excluded from Table 4 since the association were not significant

## Discussion

Malnutrition has traditionally been considered a significant health concern primarily in developing countries [31]. Although older adults comprise a significant proportion of the population in these countries, focus on their nutrition is often overlooked [8]. This research aimed to identify the factors associated with the nutritional status of the older population in Bangladesh and revealed 25.6% of the them were malnourished, which corresponds with the findings from other studies conducted in Bangladesh by Ferdous et al. (2009) and Kabir et al. (2006), where they reported the proportion of malnutrition as 26.0 and 25.8%, respectively. Studies conducted in India [32, 33] and Nepal [2, 34] showed a similar proportion of malnourishment. In contrast, a much lower proportion of malnutrition was observed in a study conducted in Hong Kong, where only 1.1% were malnourished [8]. The difference can be explained by the presence and execution of guidelines for the nutritional requirement of the older population in Hong Kong. A global study with data from community-dwelling older people of developed countries such as Switzerland, France, Japan, Sweden, and South Africa showed only 5.8% were malnourished [35]. Better healthcare facilities, especially targeting the older age group, and the existence of nutritional guidelines, which were strictly followed in the above-mentioned countries, might be the reason behind the lower proportion of malnutrition.

In the current study, the prevalence of malnutrition was higher in females (28.8%) than those in males (22.0%). A previously conducted study in Bangladesh also reported similar findings where the proportion of malnourished females and males were 29.0 and 22.0%, respectively [7]. A higher proportion of female malnourishment was also reported in studies from India and Nepal, where they reported the proportion of malnourished females as 25.3% (vs. 21.2% for males) and 31.6% (vs. 18.8% for males), respectively [2, 32].

It is suggestive that malnutrition and depression are intermingled with each other as they are two of the most common health issues encountered among this age group [36]. A significant association was observed between poor mental health status and nutritional status of the current subjects. Compared to the respondents with normal mental health status, the odds of being malnourished was 15.6 among depressed individuals. Studies conducted in Iran [37], Brazil [11], and Japan [38] showed the odds of being malnourished among depressed older people as 15.5, 4.4 and 6.3, respectively, than those with normal mental health, which corresponds with the current study finding.

In several studies, a correlation between the effects of oral health on the nutritional wellbeing of the older population was identified using the GOHAI questionnaire [39, 40]. In this current study, malnourishment was found significantly associated with poor oral health quality. Among respondents with poor oral health quality, higher odds of being malnourished was present than those of good oral health quality. Similar association was also reported by studies conducted in Brazil [41], Malaysia [42], and Lebanon [43], where they found the odds of malnutrition among the people with poor oral health quality were 3.4, 2.3, and 2.8, respectively.

The respondents’ lower educational status was another important factor for malnutrition of the older population, where the odds of having malnutrition was 6.5 among the subjects with no formal education than those of secondary education and above. Previous studies conducted by Ferdous et al. *(2009)* and Krishnamoorthy et al. *(2018)* showed 0.15 and 0.55 times less odds of being malnourished, respectively, among the respondents who had higher educational status. Although presented inversely, these study findings correspond with the current one.

The principal strength of this study was the utilization of well-validated, frequently used scales for nutritional-, mental health-, and oral health assessment, which are recognized globally.

Certain limitations are also applicable to this study. The cross-sectional study design used in this study was not ideal for identifying the cause-and-effect relationship between malnutrition and the associated factors. The sample size of this study was relatively smaller than studies of similar nature. As the purposive sampling technique was used, there was a chance of researcher bias in this study. The tools used in the data collection were not validated methodologically. Information related to food diary, biological parameters and laboratory investigations were not collected to see other determining factors of malnutrition. Generalization of the results is difficult as the study findings showed only a snapshot of the nutritional status among the older population from selected areas of Bangladesh.

## Conclusions

The majority of the older population were malnourished or at risk of malnutrition, where females were suffering more. Depression, poor oral health, and lack of education were the factors associated with the proper nutrition of the older population. To reduce malnutrition in this age group, nutrition intervention programs with special emphasis on the female is recommended. Mental and oral health should be improved for the older population to reduce the risk of malnutrition.

## Data Availability

The analyzed dataset during this study is not publicly available due to participants’ confidentiality and privacy issues but is available from the corresponding author on reasonable request.

## Abbreviations

BSMMU: Bangabandhu Sheikh Mujib Medical University
CI: Confidence Interval
COPD: Chronic Obstructive Pulmonary Disease
DPHI: Department of Public Health and Informatics
GDS-SF: Geriatric Depression Scale-Short Form
GOHAI: Geriatric Oral Health Assessment Index
MI: Myocardial Infarction
MNA: Mini Nutritional Assessment
NIPORT: National Institute of Population Research and Training
OR: Odds Ratio
PPV: Positive Predictive Value
SD: Standard Deviation
